# microRNAs targeting DEAD-box helicases are involved in salinity stress response in rice (*Oryza sativa* L.)

**DOI:** 10.1186/1471-2229-12-183

**Published:** 2012-10-08

**Authors:** Anca Macovei, Narendra Tuteja

**Affiliations:** 1 Plant Molecular Biology Group, International Centre for Genetic Engineering and Biotechnology, Aruna Asaf Ali Marg, New Delhi, 110067, India

**Keywords:** microRNAs, Helicases, Salinity stress, Experimental validation, Expression profiles

## Abstract

**Background:**

Rice (*Oryza sativa* L.), one of the most important food crop in the world, is considered to be a salt-sensitive crop. Excess levels of salt adversely affect all the major metabolic activities, including cell wall damage, cytoplasmic lysis and genomic stability. In order to cope with salt stress, plants have evolved high degrees of developmental plasticity, including adaptation via cascades of molecular networks and changes in gene expression profiles. Posttranscriptional regulation, through the activity of microRNAs, also plays an important role in the plant response to salinity conditions. MicroRNAs are small endogenous RNAs that modulate gene expression and are involved in the most essential physiological processes, including plant development and adaptation to environmental changes.

**Results:**

In the present study, we investigated the expression profiles of osa-MIR414, osa-MIR408 and osa-MIR164e along with their targeted genes, under salinity stress conditions in wild type and transgenic rice plants ectopically expressing the *PDH45* (Pea DNA Helicase) gene. The present miRNAs were predicted to target the *OsABP* (ATP-Binding Protein), *OsDSHCT* (DOB1/SK12/helY-like DEAD-box Helicase) and *OsDBH* (DEAD-Box Helicase) genes, included in the DEAD-box helicase family. An *in silico* characterization of the proteins was performed and the miRNAs predicted targets were validated by RLM-5′RACE. The qRT-PCR analysis showed that the *OsABP*, *OsDBH* and *OsDSHCT* genes were up-regulated in response to 100 and 200 mM NaCl treatments. The present study also highlighted an increased accumulation of the gene transcripts in wild type plants, with the exception of the *OsABP* mRNA which showed the highest level (15.1-fold change compared to control) in the transgenic plants treated with 200 mM NaCl. Salinity treatments also affected the expression of osa-MIR414, osa-MIR164e and osa-MIR408, found to be significantly down-regulated, although the changes in miRNA expression were limited.

**Conclusions:**

Osa-MIR414, osa-MIR164e and osa-MIR408 were experimentally validated for the first time in plants as targeting the *OsABP*, *OsDBH* and *OsDSHCT* genes. Our data showed that that the genes were up-regulated and the miRNAs were down-regulated in relation to salt stress. The negative correlation between the miRNAs and their targets was proven.

## Background

Salinity stress negatively affects the quality and quantity of crop production. Rice is considered to be a salt-sensitive crop, and excess salt levels adversely affect all the major metabolic activities, causing cell wall damage, cytoplasmic lysis or genomic instability [1]. In order to cope with salt stress, plants have evolved high degrees of developmental plasticity, including adaptation via cascades of molecular networks [2] and changes in gene expression profiles [3]. Post-transcriptional regulation also plays an important role in the plant response to salinity conditions [4].

The microRNAs (miRNAs) are a large family of endogenous, non-coding, small RNAs (21–24 nucleotides in length) that were demonstrated to play crucial roles in the modulation of gene expression [5]. Plant miRNAs originate from hairpin precursors (pre-miRNA) after two sequential cleavages performed by DICER-LIKE1 (DCL1). Mature miRNAs are then incorporated into the RISC complex (RNA-induced silencing complex) and negatively regulate the expression of specific mRNA targets through mRNA cleavage, decay or translational repression by base-pairing with its targets [6]. The first miRNA (lin-4) discovered in 1993 was reported to be involved in the regulation of *Caenorhabditis elegans* larval development [7]. Since then, an increasing interest has been devoted for the study of miRNAs both in animals and plants. After the first identification of miRNAs in *Arabidopis*[8], a great number of plant miRNAs have been identified by computational means [9]. Many miRNA genes are conserved in rice and *Arabidopsis*, indicating that their origin forfeit the divergence between monocotiledonate and dicotiledonate plants. In plants, miRNAs generally interact with their targets through near-perfect complementarity and leads to target mRNA cleavage. The high degree of sequence complementarity between miRNAs and their target mRNAs allowed the prediction of targets using algorithms that scan the genome for mRNA-miRNA complementarity [10]. However, the predicted targets must be experimentally validated in order to confirm their function.

Plant miRNAs have been predicted or confirmed to regulate a wide variety of developmental and physiological processes, such as cell proliferation, plant development, signal transduction, stress response and pathogen invasion [11]. Recent findings have demonstrated that many plant miRNAs can be induced by biotic or abiotic stress and they may play an important role in the process of adaptation to adverse environmental conditions [12,13]. For example, miR398 and miR408 were up-regulated in response to water deficit in *Medicago truncatula*[12], miR393 was found to be strongly up-regulated by cold, dehydration, NaCl and ABA treatments, while miR389a proven to be down-regulated in response to these treatments in *Arabidopsis* plants [14]. In *Vitis vinifera*, miR171e was demonstrated to be involved in the resistance to blight while the *Arabidopsis* miR399 was induced by low-phosphorus, and miR160 and miR167 are UV-B-responsive [15].

In rice, a set of miRNAs that putatively control different helicase genes were recently identified by *in silico* analysis [16]. Helicases are enzymes that catalize the separation of double-stranded nucleic acids in an energy-dependent manner. Among helicases, the DEAD-box family is by far the largest family and it is characterized by the presence of nine conserved motifs that were demonstrated to be involved in the ATPase and helicase activities [17]. Helicases are implicated in a wide range of processes such as recombination, replication and translation initiation, double-strand break repair, maintenance of telomere length, nucleotide excision repair, cell division and proliferation [18]. Recent reports indicate that the expression of genes encoding helicases is also regulated in response to changes in specific environmental conditions, including temperature, light, oxygen or osmolarity [19,20]. For example, the ectopical expression of PDH45 (Pea DNA Helicase 45) helicase, a member of the DEAD-box protein family, resulted into salinity stress tolerance both in tobacco and rice plants [21-23]. *PDH45* gene was shown to confer high salinity stress tolerance also in bacteria (*Escherichia coli*, BL2 cells) [24].

In the present study, the expression profiles of osa-MIR414, osa-MIR408 and osa-MIR164e along with their targeted genes, were investigated in relation with the early response to salinity stress. The present miRNAs were predicted to target the putative helicases *OsABP* (ATP-Binding Protein), *OsDSHCT* (DOB1/SK12/helY-like DEAD-box Helicase) and *OsDBH* (DEAD-Box Helicase) included in the DEAD-box family. No information concerning the activity of these helicases are currently available in plants. An *in silico* characterization of the proteins was performed and the miRNAs predicted targets were validated by RLM-5′RACE. Wild type and transgenic rice plants ectopically expressing the *PDH45* gene were used in the present study in order to assess if there are differences between sensitive and tolerant rice plants to early salinity stress conditions.

## Methods

### Plant materials and treatments

Rice (*Oryza sativa* subsp*. indica*) cultivar IR64 was used in the present study. Transgenic rice plants ectopically expressing the *PDH45* gene, as well as the empty vector control were raised as previously described [23]. Seeds were sowed in pots containing a mixture of vermiculite, sand, and peat moss in 1:1:1 ratio and kept under greenhouse conditions (30/20°C day/night temperature and 12 h photoperiod with 75-80% relative humidity) for two weeks. In order to test the early response to salt stress, rice plants were dipped in 100 and 200 mM NaCl solution for 3 and 6 h, respectively.

### Leaf disk assay

Leaf disks were excised from wild type, empty vector control and transgenic plants ectopically expressing the *PDH45* gene, grown for two weeks in greenhouse and submitted to salinity stress treatments. The disks were floated in 5 ml solution of NaCl (100 and 200 mM) or water as control for 72 h and the chlorophyll contend was measured. The leaf disks were homogenized using a pestle and mortar in 3 mL of 80% acetone. The homogenate was centrifuged (1,957 *g*) and the resulting supernatant was used for chlorophyll estimation. Absorbance of the solution was measured at 645 and 663 nm, using an Ultraspec 2100 pro spectrophotometer (Amersham Biosciences). Chlorophyll *a*, chlorophyll *b* and total content were calculated according to the following formulas: C_a_ = 12.72A_663_ − 2.59A_645_, C_b_ = 22.88A_645_ − 4.67A_663_, C_T_ = 20.29A_645_ + 8.05A_663_[25].

### Proline estimation

Proline was determined according to previously described procedure [26]. Plant material (500 mg) from 14-days old untreated and treated plants were extracted with 3% aqueous 5-sulphosalicylic acid and filtered through Whatman No. 2 filter paper. Glacial acetic acid and acid ninhydrine was added to the resulting supernatant and heated at 100°C for 1h. After the reaction was terminated, toluene was added. The toluene layer was then separated and the red color intensity was measured at 520nm. The assays were done in triplicates using corrected weight calculated for the actual moisture content of tissue at each treatment [27]. Proline content was expressed as mg proline per g of fresh weight.

### RNA extraction and cDNA synthesis

Total RNA was extracted from whole plant by using TRIZOL reagent (Invitrogen) according to manufacturer’s instructions. A total quantity of 100 mg plant tissue was used. The RNA samples were treated with RNase-free DNaseI (Promega) to eliminate DNA contamination. The RNA quantification was performed using the PicoGene Spectrophotometer (Genetix Biotech). The absorbance ratios of the RNA samples at 260/280 nm and 260/230 nm were between 1.9 and 2.0. The quality of RNA samples was verified on 1% agarose gel. cDNA synthesis was performed using two different strategies. In order to study the expression levels of *OsABP*, *OsDBH* and *OsDSHCT* genes, cDNA was synthesized by using the AccuScript High Fidelity 1^st^ Strand cDNA Synthesis Kit (Aegilent Technologies, Stratagene), according to manufacturer’s instructions. As for the miRNAs expression level, cDNA was obtained by using the NCode™ VILO™ miRNA cDNA Synthesis Kit (Invitrogen) as instructed by the supplier.

### microRNA validation through 5′ RNA ligase mediated rapid amplification of cDNA ends

In order to get the cleavage transcripts, a modified procedure for RNA ligase-mediated 5′ RACE was performed using the FirstChoice RLM-RACE Kit (Ambion, Invitrogen). Total RNA (1 μg) from two weeks old rice plants, was ligated to 5′ RACE adaptor without calf intestine alkaline phosphatase treatment. The gene-specific outer primers were then used for cDNA synthesis. Initial PCR was carried out using the 5′ RACE outer primer and gene-specific outer primer. Nested PCR was then carried out using 1/50 of the initial PCR reaction, with the 5′ RACE inner primer and gene specific inner primer. The sequences of the gene-specific primers are shown in Table 1. The amplified PCR products were cloned in the pCR II – TOPO vector (Invitrogen) by using the TOPO TA Cloning Kit for Sequencing (Invitrogen), according to the supplier’s instructions. Subsequently, the purified plasmids were sequenced by Eurofins Genomics, India. Three different experiments were performed for each combination.

**Table 1:** Sequences of oligonucleotide primers

**Cleavage site mapping**^**a**^			
*OsABP*	1^st^ PCR	5′-ATCAAAATCATATCCCCTCCGC-3′	258 bp
	Nested PCR	5′-TCCTCCCTCCATACGAAAATTCTG-3′	144 bp
*OsDBH*	1^st^ PCR	5′-GAAAAGGGGAACAGTGCTAG-3′	150 bp
	Nested PCR	5′-AACCAGTTCAGCTTTGGCTGCT-3′	100 bp
*OsDSHCT*	1^st^ PCR	5′-GGCAGGTGGCAGGAAGATTATA-3′	490 bp
	Nested PCR	5′-ACATCGCCGGTCATCAATCCGACAT-3′	310 bp
**QRT-PCR for gene amplification**			
*OsABP*	Forward	5′-GGGGGAGGATGGTGAGTAAT-3′	211 bp
	Reverse	5′-CCACCACCATCATCATCAAA-3′	
*OsDBH*	Forward	5′-ACGATGGCACCTCAGAGAAT-3′	177 bp
	Reverse	5′-TGGACCAAGTGACAGATGGA-3′	
*OsDSHCT*	Forward	5′-GAGGAGGTGGAGAACACGAG-3′	237 bp
	Reverse	5′-GTAGGCCTTGGCCATCTC-3′	
*α-Tubulin*	Forward	5′-GGTGGAGGTGATGATGCTTT-3′	200 bp
	Reverse	5′-ACCACGGGCAAAGTTGTTAG-3′	
**QRT-PCR for miRNA amplification**^**b**^			
osa-MIR414	Forward	5′-TCATCCTCATCATCATCGTCC-3′	100 bp
osa-MIR164e	Forward	5′-GGGAGTTCTGTGATTGGAGAG-3′	100 bp
osa-MIR408	Forward	5′-TGGAGAAGCAGGGCACGTGA-3′	100 bp
U6	Forward	5′-CGATAAAATTGGAACGATACAGA-3′	150 bp
	Reverse	5′-ATTTGGACCATTTCTCGATTTGT-3′	

^a^ Gene-specific reverse primers used in combination with the FirstChoise RLM-RACE Kit (Ambion, Invitrogen) forward and nested primers.

^b^ Specific reverse primers for miRNAs, used in combination with the Universal qPCR forward primer provided in the NCode VILO kit (Invitrogen).

### Quantitative real-time PCR

qRT-PCR reactions were performed in a 7500 Real Time PCR System apparatus (Applied Biosystems). For the *OsABP*, *OsDBH* and *OsDSHCT* gene expression, qRT-PCR primers were design by using the GeneScript Primer Design Program ( https://www.genscript.com/ssl-bin/app/primer) (Table 1). The primer pairs were design to amplify a region containing the cleavage site of osa-miMI14, osa-MIR168e and osa-MIR408, respectively. Predicted fragment size ranged between 150 and 250 bp. The *α-Tubulin* gene was used as endogenous control. The homogeneity of *α-Tubulin* gene expression under normal and salinity stress conditions was previously tested [28]. Brilliant III Ultra-Fast SYBR Green QPCR Master Mix (Aegilent Technologies, Stratagene) was used for qRT-PCR reaction as indicated by the supplier. The amplification conditions were as follows: initial incubation step at 95°C for 10 min, denaturation step at 95°C for 15 s, annealing step at 57°C for 30 s and extension step at 72°C for 30 s, for 40 cycles. Fluorescence data was collected during the extension step and the specificity of qRT-PCR products was confirmed by performing a melting temperature analysis at temperatures ranging from 55°C to 95°C in intervals of 0.5°C. PCR fragments were run in a 2.5% agarose gel to confirm the existence of a unique band with the expected size.

In the case of miRNAs expression profiles, the EXPRESS SYBR GreenER miRNA qRT-PCR Kit (Invitrogen) was used, according to the manufacturer’s instructions. To design the miRNA-specific forward primers, the qPCR Primer Design Program provided in the NCode miRNA Database ( http://escience.invitrogen.com/ncode), was used. The Universal qPCR Primer provided in the NCode VILO kit was used as a reverse primer in the qRT-PCR reaction. The small nuclear RNA U6 was used as reference control [29]. The U6 expression was uniform under normal and salinity stress conditions (data not shown). The primer sequences are shown in Table 1. The amplification conditions were as follows: UDG incubation step at 50°C for 2 min, initial incubation step at 95°C for 2 min, denaturation step at 95°C for 15 s, annealing step at 60°C for 1 min, for 40 cycles. The melting temperature analysis (60 - 95°C) was also performed.

Quantification of gene expression was performed by using the 2^-ΔΔCt^ method [30]. All reactions were performed in triplicates and graphically represented as fold change to control. The results are shown by their mean ± standard deviation.

### Bioinformatic analysis

The genomic sequences of *OsABP* (LOC_Os06g33520), *OsDBH* (LOC_Os04g40970) and *OsDSHCT* (LOC_Os11g07500) helicases were obtained from the Rice Genome Annotation Project funded by NSF ( http://rice.plantbiology.msu.edu/). The protein domain search was performed in the NCBI Conserved Domain Database (NCBI-CDD; http://www.ncbi.nlm.nih.gov/Structure/cdd/cdd.shtml). Comparison of amino acid sequences was performed using the NCBI Blast Service ( http://blast.ncbi.nlm.nih.gov/Blast.cgi). In order to determine the predicted miRNAs, the individual gene sequences were imported in psRNA Target: A Plant Small RNA Regulator Target Analysis Server ( http://plantgrn.noble.org/psRNATarget/).

### Statistical analysis

Three replicated plants from each treatment combination were selected for analysis. Results were subjected to Analysis of Variance (ANOVA) and the means were compared by Tukey’s test, which provides confidence intervals (p-values) as test results for evaluating whether the means of data sets are different.

## Results

### Salinity stress induces modifications in chlorophyll content and proline levels in rice plants

**Figure 1:**
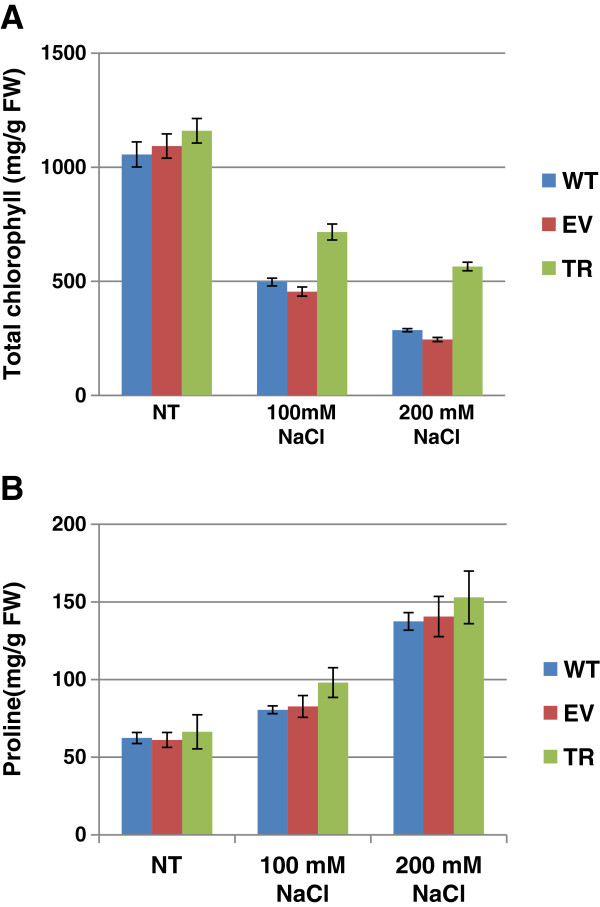
**Total chlorophyll (A) and free proline level (B) in two weeks old rice plantlets grown in greenhouse in absence/presence of salinity stress (100 and 200 mM NaCl).** WT, wild type; EV, emply vector control; TR, transgenic rice plants ectopically expressing the *PDH45* gene; NT, non-treated control.

In order to study the salt stress effects on wild type (WT), empty vector control (EV) and transgenic rice plants ectopically expressing the *PDH45* gene (TR), two different NaCl concentrations (100 mM and 200 mM) were used. The rice plants were grown under greenhouse conditions for two weeks and subsequently submitted to salt stress treatments. When leaf discs were floated separately on 100 and 200 mM NaCl for 72 h, the damage caused by salt stress was evident from the degree of bleaching observed in the leaf tissues (Additional file 1). Chlorophyll estimation was performed to confirm the leaf disc assay results. When the 100 mM NaCl solution was used, reduction in total chlorophyll content in the WT (2.1-fold), EV (2.4-fold) and TR (1.6-fold) plants, compared to the non-treated controls, was observed (Figure 1A). As for the treatment with 200 mM NaCl, the decrease in total chlorophyll content was even higher, resulting in 3.7-fold reduction in the case of WT plants, 4.1-fold for EV and 2.1-fold for TR plants.

The free proline content was also quantified, considering that this compound is a well-known osmoprotectant. The estimated free proline content in the untreated control samples was 62.4 ± 3.6 mg/g FW in WT, 61.1 ± 2.5 mg/g FW in EV and 66.4 ± 5.7 mg/g FW in TR plants. At 3 h after treatment, no significant changes in proline levels were observed (data not shown). The exposure of rice plants to salt stress for 6 h resulted into a significant (*p* < 0.001) accumulation in free proline since the 100 mM NaCl solution induced a 1.2-fold, 1.3-fold and 1.5-fold increase in the WT, EV and TR plants, compared to the untreated control (Figure 1B). A further increase in the amount of free proline was observed using the 200 mM NaCl treatments. The estimated free proline content was 137.5 ± 11.0 mg/g FW (2.1-fold compared to control) in WT, 140.6 ± 9.6 mg/g FW (2.3-fold compared to control) in EV and 159.4 ± 16.9 mg/g FW (2.4-fold compared to control) in TR plants.

The reported data show that the loss of chlorophyll induced by salinity stress was lower in TR rice plants compared to WT plants while the free proline content was slightly higher in the transgenic lines compared with the wild type plants.

### Prediction of osa-MIR414, osa-MIR408 and osa-MIR164e targets and experimental validation

The computational miRNA target searches were performed on the psRNA Target Analysis Server ( http://plantgrn.noble.org/psRNATarget/). From the bioinformatic search it was evident that the osa-MIR414, osa-MIR408 and osa-MIR164e possess multiple predicted targets. The miRNAs with their sequences, predicted targets and accession numbers are presented in Table 2. Umate and Tuteja (2010) [16] predicted that osa-MIR414 targeted six different helicases: the ATP-dependent RNA helicase DDX46 and DDX47, the Pre-mRNA-processing ATP-dependent RNA helicase PRP5, the RNA helicase A (RNAhA), an ATP-dependent helicase required to maintain repression (CTD2) and the ATP-Binding Protein (OsABP). The only predicted target that showed 100% sequence complementarity with osa-MIR414 was OsABP. The predicted targets for osa-MIR408 were DSHCT (DOB1/SK12/helY-like DEAD-box Helicase), MIP1 (Major Intrinsic Protein 1) and a basic blue copper protein (plastocyanin-like). The osa-MIR164e was predicted to target two different mRNAs: a DEAD-Box Helicase (DBH) and a member of the NAC (No Apical Meristem) gene family.

**Table 2:** Prediction of conserved miRNA targets and experimental validation by 5′-RACE

**miRNA**	**miRNA sequence**	**Predicted targets**	**Accession no**	**5′-RACE validation**
**osa-MIR414**	3′- cugcuacuacuacuccuacu – 5′	ABP	LOC_Os06g33520	Yes
		DDX46	LOC_Os08g05810	n.d
		DDX47	LOC_Os03g46610	n.d
		PRP5	LOC_Os08g06344	n.d
		RNAhA	LOC_Os01g56190	n.d
		CTD2	LOC_Os02g43460	n.d
**osa-MIR408**	3′ - cggucccuucuccgucacguc – 5′	DSHCT	LOC_Os11g07500	Yes
		MIP1	LOC_Os10g41041	n.d
		Plastocyanin	LOC_Os02g43660	Yes [12,31]
**osa-MIR164e**	3′ - agugcacgggacgaagaggu – 5′	DBH	LOC_Os04g40970	Yes
		NAC	LOC_Os06g23650	Yes [32]

**Figure 2:**
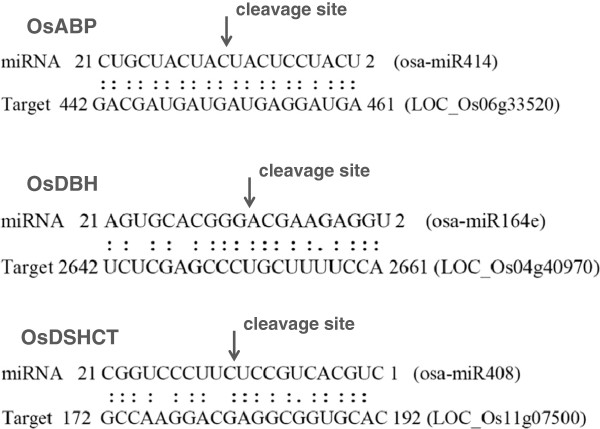
**Target validation of conserved *Oryza sativa *miRNAs.** Experimental validation of miRNA-mRNA cleavage site was performed using a modified 5′RACE assay. Each miRNA is shown base-paired to its predicted target mRNA. The sequences of miRNAs are shown in 3′ to 5′ direction. The two points indicate a Watson-Crick base-pair, one point indicates a Wooble base-pair and the space indicates a mismatch. The arrow above the target mRNA indicates the 5′ end of cleavage products. Three different experiments were performed for each combination.

To determine if the miRNAs can direct cleavage of their predicted helicase targets, a modified 5′ rapid amplification of cDNA ends (5′-RACE) protocol was performed. This technique has been used to validate miRNA targets as cleavage occurs opposite to around the tenth position from the 5′-end of the miRNA [31]. The results presented in Figure 2 indicate that the 5′-RACE analysis could clearly identify the miRNA-mRNA cleavage for the osa-MIR414 target OsABP, the osa-MIR408 target OsDSHCT and the osa-MIR164e target OsDBH in the canonical 10^th^-11^th^ positions.

### OsABP, OsDBH and OsDSHCT are putatively DEAD-box helicases

No information on *OsABP*, *OsDBH* and *OsDSHCT* are currently available. Their helicase activity was not yet validated. The only information available come form bioinformatic approaches. The OsABP (LOC_Os06g33520), OsDBH (LOC_Os04g40970) and OsDSHCT (LOC_Os11g07500) nucleotide and amino acid sequences were obtained from the Rice Genome Annotation Project funded by NSF. The *OsABP* gene contains an open reading frame (ORF) of 2772 nt, encoding a protein of 923 aa, *OsDBH* possesses an ORF of 2781 nt that encodes for a protein of 926 aa, while *OsDSHCT* is characterised by an ORF (3012 nt) encoding a protein of 1003 aa. The search for protein domains, carried out using the CDD software from NCBI, revealed the presence of the DEAD and Helicase C-terminal domains in all the three sequences (Additional file 2). The DEAD domain, specific to the DEAD-like helicase superfamily, contains several ATP-binding sites and it is involved in ATP-dependent RNA or DNA unwinding. The helicase superfamily C-terminal domain is associated with DEXD-, DEAD- and DEAH-box proteins. This domain is found in a wide variety of helicases and helicase related proteins and it is characterized by the presence of a P-loop containing Nucleoside Triphosphate Hydrolases. Members of the P-loop NTPase domain superfamily are defined by a conserved nucleotide phosphate-binding motif, also referred as Walker A and Walker B motif that bind the beta-gamma phosphate moiety of the bound nucleotides and the Mg^2+^ cation [33].

Additionally, the OsDSHCT protein included a RNA-processing arch domain and the DUF1181/NUC185 domain (Additional file 2). The RNA-processing arch domain, specific to DOB1 and SKI2 helicases, is required for the proper processing of 5.8S rRNA and it appears to function independently of canonical helicase activity [34]. The DUF1181/NUC185 domain is also found in DOB1/SK12/helY-like DEAD box helicases, involved in 3′ end formation of rRNA and mRNA transport [35].

Comparison of amino acid sequences was performed using the NCBI BlastP Service (Additional file 2). The rice OsABP protein shared 48% similarity with the *A. thaliana* DEAD-box ATP-dependent RNA helicase 31 (NP_201168) and approximately 50% similarity with the *Glicyne max* (XP_003548422) and *Vitis vinifera* (XP_002277120) proteins. The OsDBH protein revealed 50% similarity with the ATP-dependent DNA helicase Q-like 5-like form *A. thaliana* (NP_174109) and 53% similarity with the proteins from *G. max* (XP_003532718) and *V. vinifera* (XP_002266225). As for the OsDSHCT protein, 75% and 80% similarity to the *A. thaliana* (NP_565338) and *V. vinifera* (CBI24057) RNA helicase DOB1/SKI2 proteins was observed.

### Expression profiles of *OsABP*, Os*DBH* and Os*DSHCT* genes in response to early salinity stress

**Figure 3:**
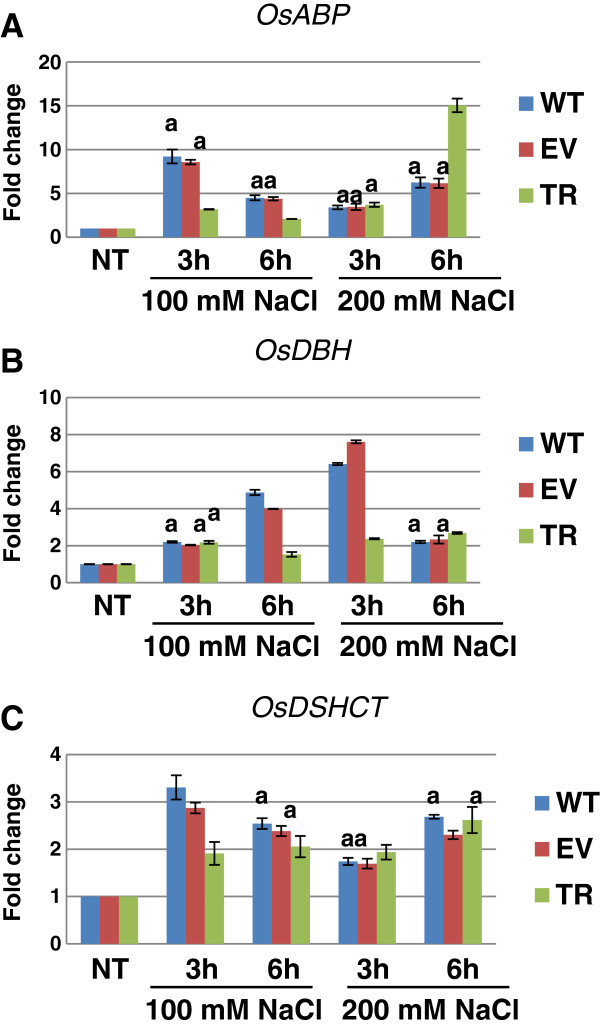
**Expression levels of *OsABP *(A), *OsDBH *(B) and *OsDSHCT *(C) genes in wild type (WT), empty vector control (EV) and transgenic rice plants ectopically expressing *PDH45 *gene (TR), grown in greenhouse and challenged with 100 and 200 mM NaCl for 3 and 6 h, respectively.** The relative expression is presented as fold change to control. Values are expressed as means ± SD of three independent replicated plants; Tuckey test *p* > 0.001 between treatments; a, no statistical differences between the samples inside each treatment; NT, non-treated control.

The expression profiles of *OsABP*, *OsDBH* and *OsDSHCT* genes were evaluated in two weeks-old rice plantlets subjected to 100 and 200 mM NaCl treatments in order to determine the early response (3 and 6 h) to salt stress conditions. The investigation was carried out using the wild type rice cultivar IR64 (WT) and the empty vector control (EV) that were compared with transgenic plants ectopically expressing the PDH45 helicase (TR). The TR plants, previously demonstrated to be tolerant to salinity stress, were used to estimate the potential differences between the mRNA levels of the *OsABP*, *OsDBH* and *OsDSHCT* genes. The results of the qRT-PCR analysis are shown in Figure 3. All the tested genes were significantly (*p* < 0.001) up-regulated in response to treatments.

The *OsABP* gene was highly up-regulated at 3 h after treatment when 100 mM NaCl was used. The WT and EV plants showed 8.2- and 7.7-fold up-regulation, while in the TR plants the observed up-regulation of the *OsABP* gene was only 2.2-fold. At 6 h after treatment the level of *OsABP* mRNA level decreased in the TR plants, compared to controls. When the 200 mM NaCl concentration was used, a change in the expression profile was observed. At 3 h after treatment only a slight increase (2.4-, 2.5- and 2.7-fold, respectively) in the *OsABP* mRNA levels was detected, while at 6 h the highest expression level (15.1-fold change compared to control) was evidenced in TR plants (Figure 3A).

When the *OsDBH* gene was analysed, the 100 mM NaCl treatment induced a slight increase in the transcript levels at 3 h following treatment was detected in all the tested samples while a consistent accumulation was evident in WT and EV plants at 6 h (3.9- and 2.9-fold, respectively). The TR plants revealed only a 0.5-fold increase in the *OsDBH* transcript level. When the 200 mM NaCl solution was used, the highest transcript accumulation was observed at 3 h after treatment in WT and EV plants (5.4- and 6.6-fold, respectively), while after 6 h the *OsDBH* transcript levels decreased (1.2- and 1.3-fold, respectively). The TR plants showed similar expression patterns (1.4- and 1.7-fold up-regulation, respectively) at both time points (Figure 3B).

As for the expression levels of the *OsDSHCT* gene, the 100 mM NaCl treatment induced a 2.3-fold increase in the transcript level in WT plants at 3 h after treatment and only 0.9-fold increase in TR plants. At 6 h after treatment, the amount of *OsDSHCT* mRNA decreased in WT plants (1.5-fold) and slightly increased in the TR plants (1.1-fold). When the 200 mM NaCl solution was used, only a slight accumulation in the transcript level was observed both at 3 h (0.7-, 0.7- and 0.9-fold) and 6 h (1.7-, 1.3- and 1.6-fold) in all the tested samples (Figure 3C).

The reported data show enhanced accumulation of the *OsABP*, *OsDBH* and *OsDSHCT* gene transcripts in WT plants, with the exception of the *OsABP* mRNA that peaked in TR plants treated with 200 mM NaCl.

### Expression analysis of osa-MIR414, osa-MIR408 and osa-MIR164e under salinity stress

**Figure 4:**
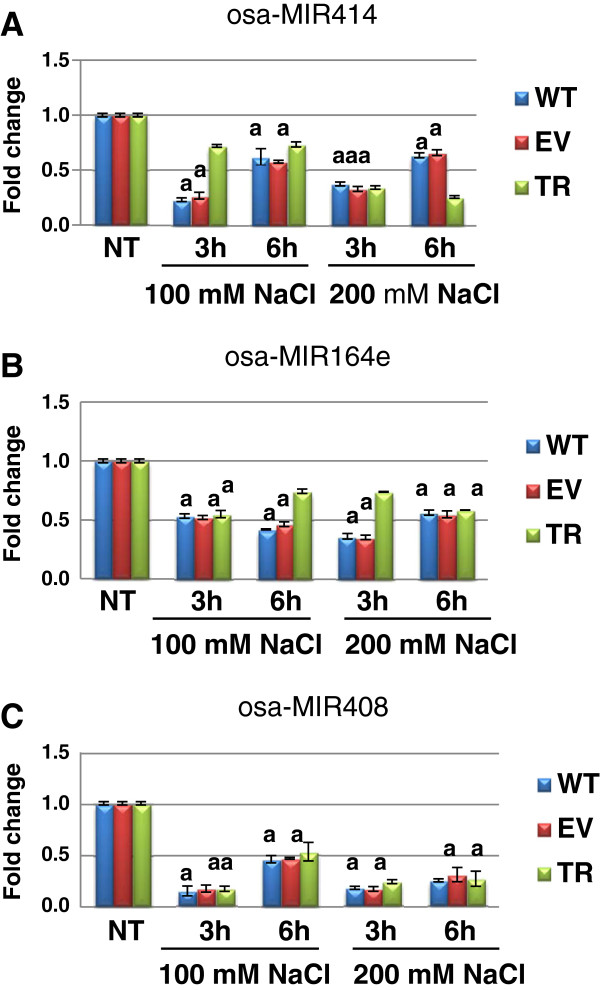
**Expression levels of osa-MIR414 (A), osa-MIR164e (B) and osa-MIR408 (C) in wild type (WT), empty vector control (EV) and transgenic rice plants ectopically expressing *PDH45 *gene (TR), grown in greenhouse and challenged with 100 and 200 mM NaCl for 3 and 6 h, respectivelly.** The relative expression is presented as fold change to control. Values are expressed as means ± SD of three independent replicated plants; Tuckey test *p* > 0.05 between treatments; a, no statistical differences between the samples inside each treatment; NT, non-treated control.

Salinity treatments also affected the expression of the miRNAs in rice plants. The results of the qRT-PCR analysis are shown in Figure 4. Osa-MIR414, osa-MIR164e and osa-MIR408 were significantly (*p* < 0.05) down-regulated in response to salinity stress.

The expression of osa-MIR414 significantly decreased (0.8-fold) in WT and EV plants at 3 h after treatment with 100 mM NaCl while in TR plants, only a 0.3-fold decrease was observed under the same conditions. At 6 h after treatments, a 0.4- and 0.3-fold decrease in the amount of osa-MIR414 was observed in WT and TR plants, respectively. Exposure to 200 mM NaCl caused a decrease of approximately 0.6- and 0.3-fold in the level of osa-MIR414 transcript after 3 and 6 h, respectively, while in the TR plants the observed reduction was around 0.8-fold (Figure 4A).

In the case of osa-MIR164e expression, the use of the 100 mM NaCl solution resulted into 0.5-fold decrease at 3 h after treatment and 0.6-fold decrease at 6 h after treatment. In the TR plants the estimated decrease of osa-MIR164e transcript was approximately 0.3-fold. When 200 mM NaCl treatment was used, a 0.7-fold decrease was measured in WT plants at 3 h after treatment, while at 6 h after treatment the WT, ER and TR plants showed a similar expression level, with a 0.4-fold reduction compared to the control (Figure 4B).

As for the osa-MIR408 expression, the amount of mRNA decreased (approximately 0.9-fold) in WT, EV and TR plants at 3 h after treatment with both the 100 and 200 mM NaCl solutions. In this case the transcript levels were similar in WT, EV and TR plants also at 6 h after treatment, when a 0.5- and 0.7-fold reduction was evidenced in response to 100 and 200 mM NaCl, respectively (Figure 4C).

Although salinity treatment affected the expression of all tested miRNAs, the reported changes in miRNA levels were small.

## Discussions

In the present study, transgenic rice plants ectopically expressing the *PDH45* gene (TR), were tested under early-induced salinity stress conditions and compared with the wild type rice genotype IR64 (WT) and the empty vector control (EV). The TR plants are resistant to salinity stress and represent a valid system for testing the genes and miRNAs expression patters in comparison with the WT plants. The short-term response to salinity stress may be particularly relevant to better understand the biological significance of stress status in plant responses to salinity.

A preliminary characterization of the stress response in rice was carried out by measuring the chlorophyll content and the free proline levels. The damage caused by salt stress was evident from the degree of bleaching observed in the leaf tissues, along with a decrease in chlorophyll content. However, the reduction in chlorophyll content was significantly higher in WT plants, compared with TR plants. Since the goal of this study was to test the plant response to early-induced salinity stress, the free proline levels were measured at 3 and 6 h post-treatment. Significant increase in free proline was observed in response to both NaCl concentrations (100 and 200 mM) at 6 h after treatment. The TR plants were able to accumulate higher amounts of free proline compared with the WT. This finding is in agreement with the recent study on rice plants ectopically expressing the *PDH45* gene, where the authors demonstrated that the plants were tolerant to salinity stress [22,23].

The role of miRNAs as important players in the plant stress response has been highlighted in recent years, opening new intriguing perspectives for crop improvement.

The main goal of the present work was to assess the role of three different rice miRNAs, osa-MIR414, osa-MIR164e and osa-MIR408, as regulators of genes encoding DEAD-box helicases in response to salinity stress. The miRNA hereby investigated have been described in a previous work [16] where an *in silico* analysis was used to identify a set of miRNAs involved in the regulation of rice helicases expression. A modified RLM -5′RACE protocol was used to validate osa-MIR414, osa-MIR164e, and osa-MIR408 predicted targets and our results clearly demonstrate that osa-MIR414 targets the OsABP mRNA, osa-MIR408 targets OsDSHCT and osa-MIR164e targets the OsDBH mRNA sequence, cleaving them in the canonical 10^th^-11^th^ positions. In plants, there are only few reports concerning miRNAs that target specific helicase genes. In a recent study it demonstrated that the DCL1 helicase domain, targeted by miR168, is responsible for the ATP-dependence of miRNA processing, suggesting that this helicase domain is critical for miRNA biogenesis [36]. Besides the *OsABP*, *OsDBH* and *OsDSHCT* DEAD-box helicase genes, other predicted targets of the above cited miRNAs have been reported, as shown in Table 2. Some of these targets were already validated while others still need to be studied. Out of the six helicase that were predicted to be targets of osa-MIR414, only the OsABP protein was experimentally validated in this study. In the case of osa-MIR408, the predicted target corresponding to a plastocyanin-like protein has been previously validated in *Arabidposis*[31] and *Medicago truncatula*[12]. Other miR408 targets include some members of Major Intrinsic Proteins (MIPs) involved in plant osmoregulation, drought resistance, salt tolerance and seed germination [37]. In this case, the computational predicted target was not yet validated. As for osa-MIR164, the subset of the *Arabidopsis* NAC-domain transcription factor genes that include the CUP-SHAPED COTYLEDON (*CUC1*, *CUC2* and *CUC3*) genes were already validate as targets [32]. The NAC-domain genes are required for organ separation. When miR164 was ectopically expressed in *Arabidopsis* plants, it resulted into floral organ fusion and cotyledon fusion [38].

No information regarding the activity of OsABP, OsDBH and OsDSHCT helicases are currently available in plants. In consequence, a thorough bioinformatic investigation was carried out to analyze and compare in detail the structural features of the OsABP, OsDBH and OsDSHCT proteins. The three proteins harbour the DEAD domain and the helicase C-terminal domain, typically conserved motifs found in helicase superfamily. The similarity search showed that OsABP and OsDBH were close to RNA helicase 31 and DNA helicase RecQl5, respectively, while the OsDSHCT revealed high similarity with the SKI2/DOB1 protein. RNA helicase 31 is a member of the DEAD-box family, possessing ATP-dependent helicase activity and RNA-binding property [33]. The RecQ helicases have been shown to play important roles in DNA repair, recombination and replication, consistent with their intrinsic DNA helicase activities [39]. Recently it was proved that the RecQl5 helicase promotes genome stabilization through interaction with RNA polymerase II [40]. The SKI/DOB1 proteins are essential ATP-dependent RNA helicases that play crucial roles in ribosome synthesis and the degradation of cytoplasmic mRNAs following poly (A) [41]. A recent report on OsABP revealed that the gene is up-regulated in response e to multiple abiotic stress treatments including NaCl, dehydration, ABA, blue and red light [42]. Although a large number of DEAD box proteins were identified by *in silico* analysis as putative predicted helicases, the confirmation of helicase activity was demonstrated only for few of them [33]. In plants, two DEAD-box-related helicases termed PDH47 and PDH45 were proven to be induced by a variety of abiotic stresses including salinity, cold, heat and ABA treatments, suggesting that they are components of a general stress response mechanism [21-23,43]. Recently, a single subunit of minichromosome maintenance 6 (MCM6) from pea was reported to function as DNA helicase and to promote salinity stress tolerance without affecting yield [44-46]. Nonetheless the few references, genes encoding helicases were proven to be induced in response to salinity stress [47].

The stress-responsive gene expression results in metabolites accumulation, alteration of biochemical and physiological pathways, vital for the plant adaptation to stress conditions [14]. In the present study, qRT-PCR analysis showed that the *OsABP*, *OsDBH* and *OsDSHCT* genes are up-regulated in response to salinity stress in both WT and TR plants. However, a higher accumulation of the gene transcripts was observed in WT plants, except for the *OsABP* transcript that showed the highest level in TR plants treated with 200 mM NaCl. The differences observed between the experimental conditions and the control might be due to changes in transcriptional activity. However, some differences might result from the action of miRNAs [48]. The effects of miRNA directed mRNA degradation can be detectable through changes in the expression profiles of miRNA targeted genes. Our results shown that osa-MIR414, osa-MIR164e and osa-MIR408 were down-regulated in WT and TR rice plants challenged with salinity stress. Considering the fact that the targeted helicases were up-regulated and the miRNAs were down-regulated in relation to early-induced salt stress, we can state that the negative correlation between these miRNAs and their validated targets is certified. The levels of accumulation or decrease in the transcript levels is most visible in the case of TR plants, where the 0.9-fold decrease in the osa-MIR414 expression resulted into 15.1-fold up-regulation of *OsABP* gene.

Several recent papers have described the miRNAs involvement in the response to various stress conditions [49]. miR408 was shown to accumulate at low levels in plants under standard conditions and its accumulation was highly induced by drought and heavy-metals [31,32]. Since miRNAs can be targeted by more than one gene, a recent study demonstrated that AGO1 and AGO2 could act redundantly in the miR408-mediated plastocyanin regulation [50]. The *Arabidopsis* miR164 family comprises three members, miR164a, miR164b and miR164c, which negatively regulate several genes that encode NAC-like transcription factors [32]. In the present study, osa-MIR164e was identified and its target, a DEAD-box helicase, validated. Both osa-MIR408 and osa-MIR164e are responsive to early salinity stress conditions. miR164 and miR408 were proven to be responsive also to cold and drought conditions in Euphorbiaceae plants [10]. Several miRNAs that displayed different activities during salt stress were recently identified in *Zea mais*[51]. The authors shown that miR164 was down-regulated after 24 h of salt shock, while its target NAC1 (an early auxin-responsive gene) was up-regulated. A similar study showed that miR156, miR166, miR171, miR172, miR319, miR164 along with their target genes, were differentially expressed in stress-tolerant maize hybrids compared with stress-sensitive lines [52]. As for osa-MIR414, no other information was available in the literature. It was predicted to target six different helicases in rice plants [16], we validated the *OsABP* gene as one of its targets and shown the changes in their expression profiles under early salt stress conditions. However, further analysis is still needed in order to better characterize the miRNAs involvement in salinity stress as well as the function of their validated targets.

## Conclusions

Osa-MIR414, osa-MIR164e and osa-MIR408 were experimentally validated for the first time in plants as targeting the *OsABP*, *OsDBH* and *OsDSHCT* genes. OsABP, OsDBH and OsDSHCT represent newly identified DEAD-box helicases in rice. Their function still needs to be experimentally validated. The expression profiles of the miRNAs and their validated targets were tested under early-induced salinity stress conditions both in wild type and transgenic rice plants ectopically expressing the PDH45 helicase. Our data showed that that the genes were up-regulated and the miRNAs were down-regulated in relation to salt stress. The negative correlation between the miRNAs and their targets was proven. The present results also highlights higher accumulation of the gene transcripts in WT plants when compared with TR plant, correlated with decrease in the miRNAs expression.

## Abbreviations

miRNA: microRNA; RLM-5′RACE: RNA ligase-mediated 5′ rapid amplification of cDNA ends; qRT-PCR: quantitative Real-Time Polymerase Chain Reaction; OsABP: ATP-Binding Protein; OsDBH: DEAD-Box Helicase; OsDSHCT: DOB1/SK12/helY-like DEAD-box Helicase; PDH45: Pea DNA Helicase 45; NaCl: Sodium chloride; WT: Wild type; EV: Empty vector control; TR: Transgenic plants.

## Competing interests

Authors declare that they have no competing interests.

## Authors’ contributions

AM planned and performed all the experimental work. NT supervised the work and helped in the preparation of the final draft of the manuscript. Both authors read and approved the final manuscript.

## Supplementary Material

Additional file 1 Leaf disk assay. Click here for file

Additional file 2 Domain organization of OsABP, OsDBH and OsDSHCT proteins. Click here for file
