# Supplementary material for: microRNAs targeting DEAD-box helicases are involved in salinity stress response in rice (Oryza sativa L.)
Source: BMC Plant Biol. 2012 Oct 8;12:183. doi: 10.1186/1471-2229-12-183 (PMC3502329; doi:10.1186/1471-2229-12-183)
Supplement: Additional file 1 — Leaf disk assay. [file 1471-2229-12-183-S1.docx]

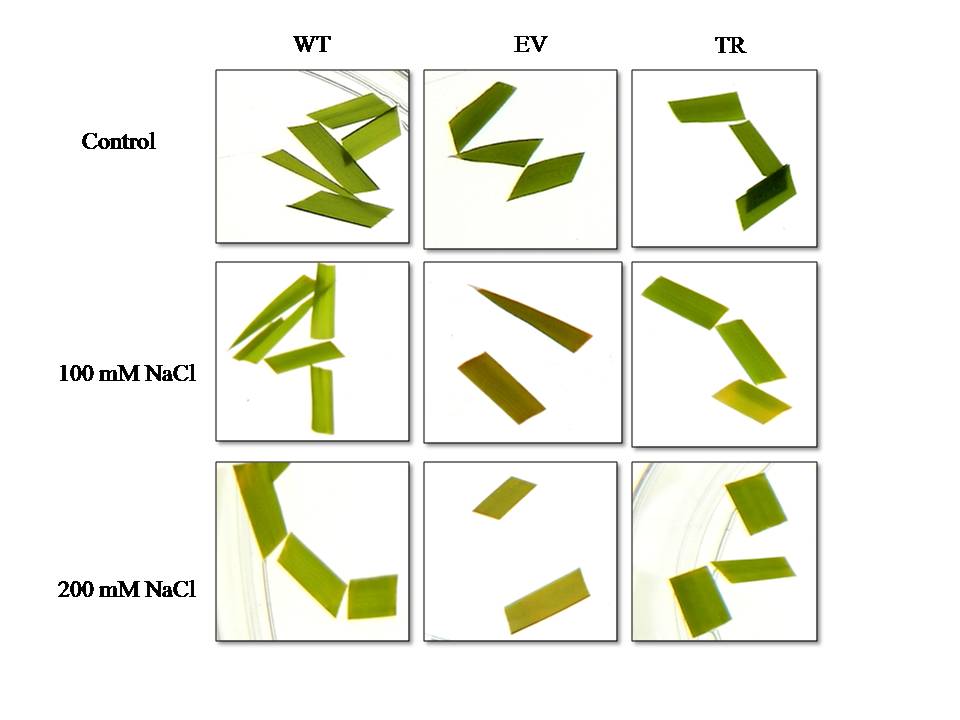


**Additional file 1:** **Leaf disk assay.** Rice leaves were excised from wild type (WT), empty vector control (EV) and transgenic plants ectopically expressing the *PDH45* gene (TR). The plants were grown for two weeks in greenhouse and submitted to salinity stress treatments. The disks were floated for 72 h in 5 ml solution of 100 and 200 mM NaCl or water as control. The damage caused by salt stress is evident from the degree of bleaching observed in the leaf tissues.
