# Supplementary material for: microRNAs targeting DEAD-box helicases are involved in salinity stress response in rice (Oryza sativa L.)
Source: BMC Plant Biol. 2012 Oct 8;12:183. doi: 10.1186/1471-2229-12-183 (PMC3502329; doi:10.1186/1471-2229-12-183)
Supplement: Additional file 2 — Domain organization of OsABP, OsDBH and OsDSHCT proteins. [file 1471-2229-12-183-S2.docx]

**A**

**B**

| **Percentage of similarity** | | | | |
| --- | --- | --- | --- | --- |
| ***O. sativa*** | ***A. thaliana*** | ***G. max*** | ***V. vinifera*** | **BLAST Hits** |
| **ABP** | 48 % | 51.5 % | 51.1 % | DEAD-box ATP-dependent RNA helicase 31 |
| **DBH** | 50 % | 53 % | 53.2 % | ATP-dependent DNA helicase Q-like 5-like |
| **DSHCT** | 75 % | - | 80 % | RNA helicase ATP-dependent SK12/DOB1 protein |

**Additional file 2: Domain organization of OsABP (LOC_Os06g33520), OsDBH (LOC_Os04g40970) and OsDSHCT (LOC_Os11g07500) proteins (A).** The *OsABP* gene contains an open reading frame (ORF) of 2772 nt, encoding a protein of 923 aa, *OsDBH* possesses an ORF of 2781 nt that encodes for a protein of 926 aa, while *OsDSHCT* is characterised by an ORF (3012 nt) encoding a protein of 1003 aa. OsABP (ATP-Binding Protein), 488-659 aa DEAD domain, 732-808 P-loop C-terminal domain; OsDBH (DEAD-Box Helicase), 287-454 aa DEAD domain, 520-596 aa P-loop C-terminal domain; OsDSHCT (DOB1/SK12/helY-like DEAD-box Helicase), 85-236 aa DEAD domain, 397-481 aa P-loop C-terminal helicase domain, 537-802 aa RNA-processing arch domain, 814-1003 aa DUF1181/NUC185 domain. **Percentage of similarity between the ABP, DBH and DSHCT proteins form rice, *Arabidopsis thaliana*, *Glycine max* and *Vitis vinifera* (B)**.
